# A Risk Model for 1-Year Mortality After Transcatheter Aortic Valve Replacement From the J-TVT Registry

**DOI:** 10.1016/j.jacasi.2022.06.002

**Published:** 2022-10-04

**Authors:** Koichi Maeda, Hiraku Kumamaru, Shun Kohsaka, Kazuo Shimamura, Isamu Mizote, Kizuku Yamashita, Ai Kawamura, Takashi Mukai, Daisuke Nakamura, Yasuharu Takeda, Hideyuki Shimizu, Yasushi Sakata, Toru Kuratani, Shigeru Miyagawa, Yoshiki Sawa

**Affiliations:** a Department of Cardiovascular Surgery, Osaka University Graduate School of Medicine, Osaka, Japan; b Department of Healthcare Quality Assessment, Graduate School of Medicine, University of Tokyo, Tokyo, Japan; c Department of Cardiology, Keio University School of Medicine, Tokyo, Japan; d Department of Cardiology, Osaka University Graduate School of Medicine, Osaka, Japan; e Department of Cardiovascular Surgery, Keio University School of Medicine, Tokyo, Japan

**Keywords:** prediction model, registry, transcatheter aortic valve replacement, AS, aortic valve stenosis, BMI, body mass index, BSA, body size area, EuroSCORE, European System for Cardiac Operative Risk Evaluation, NYHA, New York Heart Association, STS PROM, Society of Thoracic Surgeons Predicted Risk of Mortality, TAVR, transcatheter aortic valve replacement

## Abstract

**Background:**

Although transcatheter aortic valve replacement (TAVR) has demonstrated favorable outcomes in randomized studies, there remains a sizable group of patients in whom TAVR may be futile. Characterizing the survival rate in a wide array of patients undergoing TAVR can help develop effective strategies for improving the allocation of medial resources.

**Objectives:**

The aim of this study was to develop a risk model to estimate 1-year mortality after TAVR from a representative nationwide registry in Japan.

**Methods:**

The J-TVT (Japan Transcatheter Valve Therapies) registry contains complete data, including 1-year outcomes, on patients undergoing TAVR in Japan. A total of 17,655 patients underwent TAVR between 2013 and 2018. They were randomly divided into 2 groups in a 7:3 ratio to form a derivation cohort of 12,316 patients and a validation cohort of 5,339 patients. A risk model was constructed for 1-year mortality in the derivation cohort, and its discrimination and calibration were assessed in the validation cohort.

**Results:**

The mean age of all registered patients was 84.4 years, and 68.8% were women. The mean body size area was 1.43 m^2^, and the mean Society of Thoracic Surgeons Predicted Risk of Mortality score was 7.3%. The estimated 1-year survival was 91.8%; 202 and 1,316 deaths were observed at 30 days and 1 year, respectively; The estimated C index for the developed model was 0.733 (95% CI: 0.709-0.757) in the validation cohort, with good calibration.

**Conclusions:**

A prediction model for 1-year survival following TAVR derived from a national clinical database performed well and should aid physicians managing TAVR patients.

Transcatheter aortic valve replacement (TAVR) has been shown to be a safe and effective alternative to surgical aortic valve replacement in patients with severe aortic valve stenosis (AS). TAVR recently showed favorable outcomes in intermediate-risk and low-risk patients.^1^, ^2^, ^3^, ^4^ Risk assessment has become a foundational element of preprocedural evaluation in patients with AS prior to TAVR. The established scoring systems used to estimate the risk for adverse outcomes in surgical aortic valve replacement (eg, European System for Cardiac Operative Risk Evaluation [EuroSCORE], EuroSCORE II, Society of Thoracic Surgeons Predicted Risk of Mortality [STS PROM] score) provide useful reference points for weighing the procedural benefits of TAVR. However, there remain concerns regarding performing TAVR in patients who have significant cardiovascular or comorbid conditions with guarded long-term prognosis (often defined as “cohort C” patients). In general, TAVR is recommended for patients who would benefit from it in the long term and are expected to survive beyond 12 months, from the perspective of therapeutic effect and/or cost-effectiveness. Therefore, it is important to identify these patients and to allocate medical resources appropriately.

To date, several risk models have been developed to risk-stratify TAVR patients. However, most of these scoring systems provide information on short-term outcomes, including 30-day or in-hospital outcomes.^5^, ^6^, ^7^, ^8^, ^9^ However, previously reported quantitative risk assessment of long-term mortality following TAVR has been contemplated mainly using models developed from a limited number of cases at a single center or a few centers and therefore has limited generalizability.^10^, ^11^, ^12^, ^13^

An accurate large longitudinal dataset is a prerequisite for developing a long-term risk model. However, the unavailability of such a dataset has hampered model development. To the best of our knowledge, a well-accepted statistical 1-year risk model specifically designed for patients undergoing TAVR does not exist. The nationwide J-TVT (Japan Transcatheter Valve Therapies) registry was launched in 2013. J-TVT registry institutions and operators are required to consecutively register all cases and their 1-year morbidity and mortality outcomes for certification. Furthermore, institutions must confirm case registration every 3 years for renewal of institutional certification. With more than 18,000 consecutive patients registered to date, the J-TVT registry provides a unique opportunity to construct 1-year mortality risk models. The aim of this study was to develop and validate a multivariable risk model to estimate 1-year mortality after TAVR using data from the J-TVT registry.

## Methods

This study was approved by Japan Transcatheter Valve Therapies, an organization managing the data use of the J-TVT registry, and by the Institutional Review Board of the Osaka University Graduate School of Medicine (approval number 19375).

### Data source

The J-TVT registry is a nationwide database of all patients undergoing TAVR in Japan. It was developed and is governed by 4 Japanese academic societies involved in TAVR treatment (the Japanese Circulation Society, the Society of Japanese Cardiovascular Surgery, the Japanese Association for Thoracic Surgery, and the Japanese Association of Cardiovascular Intervention and Therapeutics). Facilities must be approved as TAVR implementation facilities through a rigorous selection process by the societies to receive TAVR devices from manufacturers. Facilities are also required to register all their TAVR cases as well as 30-day and 1-year outcomes in the J-TVT registry.^14^ Annual routine on-site audits (approximately 10 institutions annually) by those entrusted by the 4 Japanese academic societies are performed for data accuracy. The annual numbers of cases registered in the registry in 2014 and 2020 exceeded 1,000 and 9,000, respectively. Appropriate clinical indications for TAVR are determined by the heart team, comprising cardiovascular surgeons and cardiologists.

We identified 18,116 consecutive patients who underwent their first TAVR procedures between August 2013 and December 2018 at 131 institutions in Japan. As TAVR was considered off label for patients on long-term dialysis at the time of the study, we excluded them (n = 90 [0.5%]). We also excluded patients who had no follow-up information during the 1-year period at the time of analysis (n = 371 [2.1%]). Consequently, 17,655 patients were included in the final analysis.

### Outcome and covariates

The outcome of interest was all-cause 1-year mortality following TAVR. On the basis of prior research and expert opinion, we selected potential covariates from patients’ demographics (age, sex, body size area [BSA], and body mass index [BMI]), preprocedural status (New York Heart Association [NYHA] functional classification), comorbidities (presence of coronary artery disease, chronic obstructive pulmonary disease, hypertension, diabetes mellitus, carotid artery disease, cerebrovascular disease, peripheral vessel disease, malignancy, immunodeficiency, prior cardiovascular surgery including coronary artery bypass grafting, percutaneous coronary intervention, and severe aortic calcification [porcelain aorta]), echocardiographic findings (pulmonary hypertension, aortic and mitral insufficiency grade, bicuspid valve or tricuspid valve, left ventricular ejection fraction, mean gradient, and aortic valve area), laboratory data (hemoglobin, serum albumin, and creatinine), and procedural characteristics (whether the procedure was elective or nonelective) from the J-TVT registry data collection form. Although we did not use the STS PROM score for prediction modeling, the STS PROM score was calculated using the registered baseline covariates^15^ and was used as a comparator to evaluate the predictive performance of the model. Among the covariates included in the prediction modeling, age was missing for 31 patients (0.2%), hemoglobin for 37 patients (0.2%), serum albumin for 395 patients (2.2%), serum creatinine for 33 patients (0.2%), and left ventricular ejection fraction, mean gradient, and aortic valve area for 74 patients (0.4%).

### Statistical analysis

We tabulated patients’ characteristics using counts and percentages for categorical variables and mean ± SD for continuous variables. We assessed the incidence of mortality within 1 year after TAVR among registered patients using the Kaplan-Meier method. Patients were censored 1 year after the procedure date or at the last follow-up date registered in the database, whichever came earlier. We evaluated the crude relationship between the continuous covariates and the outcome by drawing Kaplan-Meier curves by groups categorized using cutoff points with equal intervals of these covariates.

We divided the study group into derivation and validation cohorts by randomly selecting patients in a 7:3 ratio; there were 12,316 patients in the derivation cohort and 5,339 patients in the validation cohort. We summarized STS PROM score, procedure year, and TAVR device generation for each cohort. In the derivation cohort, there were 349 patients (2.8%) with at least 1 missing data component. As a primary analysis, we constructed a multivariable Cox proportional hazards regression model with the outcome of all-cause death using the explanatory variables previously described among patients with complete data (n = 11,967 [97.2%]).

To assess the performance of the developed model, the model from the complete case analysis was then applied to the patients in the validation cohort to predict their 1-year mortality using the Breslow estimator.^16^ We evaluated the model’s discriminatory accuracy using Harrell’s C index (concordance statistic) and depicted a time-dependent receiver-operating characteristic curve at last event occurrence.^17^ Using the validation cohort, we also depicted the calibration plot for predicted vs observed 1-year mortality outcomes in 10 equally sized groups ranked by their predicted values. Observed 1-year mortality was estimated using the Kaplan-Meier method. To compare the performance of the developed model with that of an established risk score, we also assessed the discrimination performance of the STS PROM score and logistic EuroSCORE using receiver-operating characteristic curves among patients with these data available in the database.

We also conducted an additional analysis with multiple imputation in the whole cohort, using the fully conditional specification approach in the PROC MI package in SAS (SAS Institute), creating 50 datasets from all variables presented in Table 1. We constructed Cox proportional hazards models for 1-year mortality in the 50 development datasets, the estimates were pooled using Rubin’s rule, and the differences were assessed against those from the complete case analysis. The performance of the models from multiple imputation analysis were also assessed in the corresponding 50 validation datasets using the pooled C index.

**Table 1:** Baseline Patient Characteristics

	All (N = 17,655)	Derivation (n = 12,316)	Validation (n = 5,339)
Demographics and physical status			
Age, y	84.4 ± 5.2	84.4 ± 5.2	84.4 ± 5.2
<65	55 (0.3)	40 (0.3)	15 (0.3)
65-74	659 (3.7)	441 (3.6)	218 (4.1)
75-84	7,705 (43.6)	5,402 (43.9)	2,303 (43.1)
≥85	9,205 (52.1)	6,407 (52.0)	2,798 (52.4)
Missing age	31 (0.2)	26 (0.2)	5 (0.1)
Female	12,153 (68.8)	8,464 (68.7)	3,689 (69.1)
Height, cm	150.0 ± 9.3	149.9 ± 9.3	149.8 ± 9.2
Weight, kg	50.2 ± 10.5	50.2 ± 10.5	50.2 ± 10.7
BSA, m^2^	1.43 ± 0.17	1.43 ± 0.17	1.43 ± 0.17
BMI, kg/m^2^	22.3 ± 5.5	22.3 ± 6.0	22.3 ± 3.9
<18.5	2,670 (15.1)	1,863 (15.1)	807 (15.1)
≥18.5≤ to <25	11,236 (63.6)	7,862 (63.8)	3,374 (63.2)
≥25 to <30	3,204 (18.1)	2,236 (18.2)	968 (18.1)
≥30	545 (3.1)	355 (2.9)	190 (3.6)
NYHA functional class III or IV	4,576 (25.9)	3,213 (26.1)	1,363 (25.5)
STS score, %	7.3 ± 4.8	7.3 ± 4.9	7.4 ± 4.7
CAD	5,677 (32.2)	3,950 (32.1)	1,717 (32.2)
COPD (moderate or severe)	1,443 (8.2)	1,039 (8.4)	404 (7.6)
Hypertension	13,969 (79.1)	9,714 (78.9)	4,255 (79.7)
DM	5,328 (30.2)	3,162 (25.7)	1,433 (26.8)
Insulin-dependent DM	734 (4.2)	487 (4.0)	247 (4.6)
Noncardiac artery disease	2,281 (12.9)	1,589 (12.9)	692 (13.0)
Carotid artery disease	1,143 (6.5)	786 (6.4)	357 (6.7)
Cerebrovascular disease	2,125 (12.0)	1,493 (12.1)	632 (11.8)
Peripheral vessel disease	2,065 (11.7)	1,431 (11.6)	634 (11.9)
Malignancy	1,594 (9.0)	1,115 (9.1)	479 (9.0)
Immunodeficiency	631 (3.6)	454 (3.7)	177 (3.3)
Previous cardiac surgery	1,570 (8.9)	1,118 (9.1)	452 (8.5)
Previous CABG	917 (5.2)	652 (5.3)	265 (5.0)
Previous PCI	4,141 (23.5)	2,880 (23.4)	1,261 (23.6)
Pulmonary hypertension	2,742 (15.5)	1,937 (15.7)	805 (15.1)
Porcelain aorta	1,670 (9.5)	1,179 (9.6)	491 (9.2)
Hemoglobin, g/dL	11.3 ± 1.8	11.3 ± 1.8	11.3 ± 1.7
Missing hemoglobin	37 (0.2)	18 (0.1)	19 (0.4)
Albumin, g/dL	3.8 ± 1.8	3.8 ± 1.9	3.8 ± 1.6
Missing albumin	395 (2.2)	265 (2.2)	130 (2.4)
Creatinine, mg/dL	1.05 ± 1.19	1.05 ± 1.29	1.05 ± 0.95
Missing creatinine	33 (0.2)	20 (0.2)	13 (0.2)
Aortic insufficiency grade III or IV at baseline	1,474 (8.3)	1,040 (8.4)	434 (8.1)
Mitral insufficiency grade III or IV at baseline	1,443 (8.2)	1,006 (8.2)	437 (8.2)
Bicuspid AV	437 (2.5)	298 (2.4)	139 (2.6)
LVEF at baseline, %	61.5 ± 12.2	61.4 ± 12.3	61.8 ± 12.1
AV mean gradient at baseline, mm Hg	49.8 ± 18.0)	49.7 ± 17.8	49.9 ± 18.3
AV area at baseline, cm^2^	0.65 ± 0.34	0.60 ± 0.30	0.60 ± 0.30
Missing LVEF, mean gradient, and AV area	74 (0.4)	44 (0.4)	30 (0.6)
Procedural			
Elective	17,285 (97.9)	12,061 (97.9)	5,224 (97.8)
Nonelective	370 (2.1)	255 (2.1)	115 (2.2)
Approach			
Transfemoral	15,289 (86.6)	10,685 (86.8)	4,604 (86.2)
Others	2,366 (13.4)	1,631 (13.2)	735 (13.8)
Outcome			
30-d death	202 (1.1)	143 (1.2)	59 (1.1)
1-y death	1,316 (7.5)	926 (7.5)	390 (7.3)

Values are mean ± SD or n (%).

AV = aortic valve; BMI = body mass index; BSA = body surface area; CABG = coronary artery bypass grafting; CAD = coronary artery disease; COPD = chronic obstructive pulmonary disease; DM = diabetes mellitus; LVEF = left ventricular ejection fraction; NYHA = New York Heart Association; PCI = percutaneous coronary intervention; STS = Society of Thoracic Surgeons.

All analyses were performed using SAS version 9.4.

## Results

### Baseline characteristics

Among the 17,655 patients (97.9% of all first-time procedures in the database) included in the final analysis, their mean age was 84.4 years, and 68.8% were women. Notable patient characteristics included a mean BSA of 1.43 ± 0.17 m^2^ and a mean BMI of <18.5 kg/m^2^ in 15.1% of patients. In addition, 25.9% of patients were in NYHA functional class III or IV, and they were high- or intermediate-risk patients (the mean STS PROM score was 7.3% ± 4.8% overall). The characteristics of the patients were well balanced between the derivation and validation cohorts (Table 1, Supplemental Table 1).

### Outcomes

**Figure 1:**
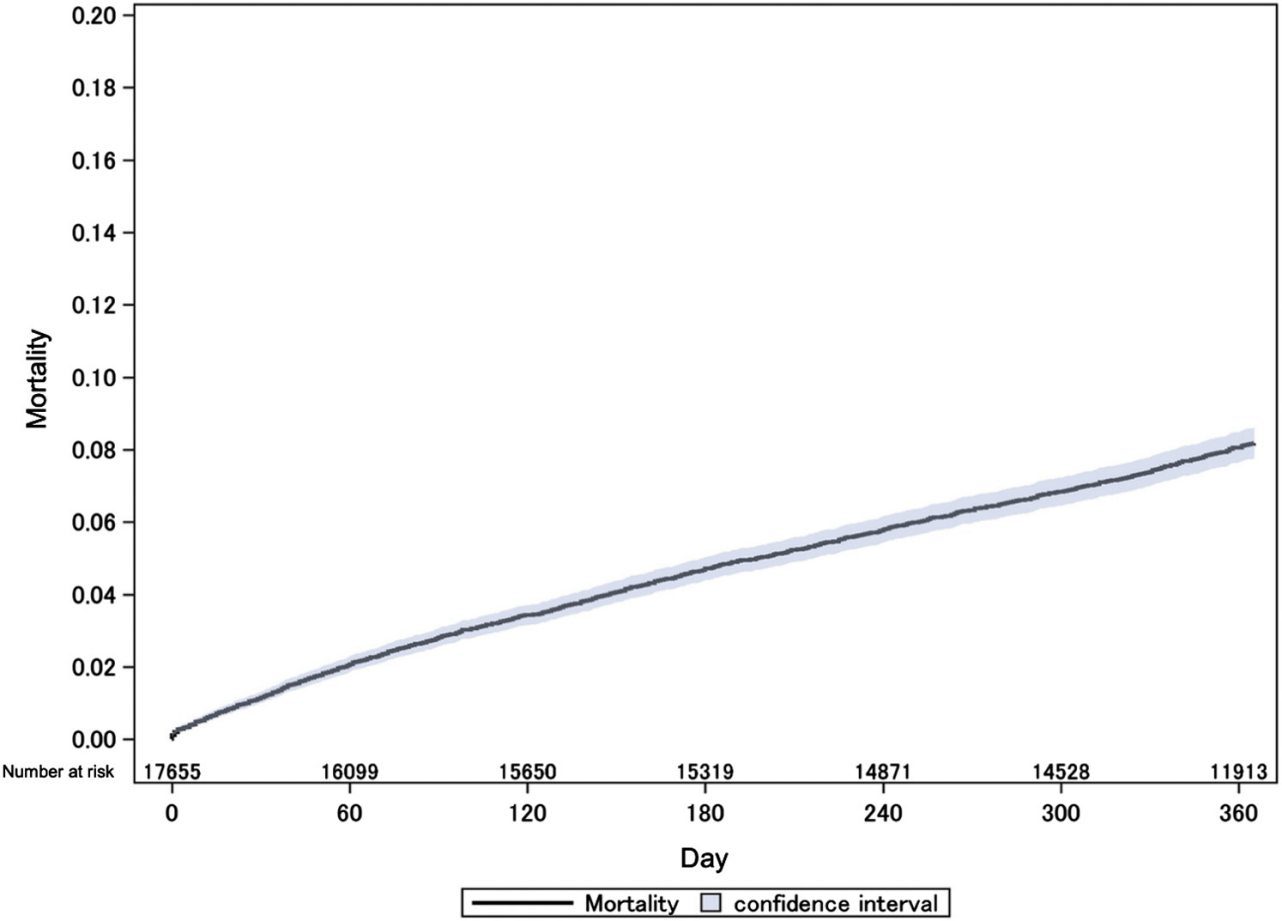
Kaplan-Meier Curve for Overall Mortality (N = 17,655) The number of events in the full cohort was 1,316, and the total observation period was 5,567,375 person-days.

Among all patients, the total follow-up duration was 5,567,375 person-days, with a median follow-up duration of 365 days. There were 1,316 deaths (incidence rate: 2.36 deaths/10,000 person-days), and 92.5% of the patients were either followed up to 365 days or had died within 1 year. Mortality rates were 1.1% and 8.2% at 30 days and 1 year, respectively (Figure 1).

In the derivation cohort, we identified 926 deaths over 3,885,025 person-days (incidence rate: 2.38 deaths/10,000 person-days). In the validation cohort, we identified 390 deaths over 1,682,350 person-days (incidence rate: 2.32 deaths/10,000 person-days).

### Results of the Cox proportional hazards model

In the derivation sample (n = 12,316), 11,967 patients had complete documentation of model predictors. Factors strongly associated with 1-year mortality included older age (≥85 years), sex (male), lower BMI, NYHA functional class III or IV, presence of chronic obstructive pulmonary disease, peripheral vessel disease, malignancy, immunodeficiency, porcelain aorta, lower hemoglobin, lower serum albumin, higher creatinine, and emergency procedures. Table 2 presents the HR estimates and their 95% CIs for the 27 variables in the model.

**Table 2:** Multivariate Logistic Regression Analysis for the Association With 1-Year Mortality After Transcatheter Aortic Valve Replacement

	Coefficient	HR (95% CI)	P Value
Age	0.02085	1.02 (1.01-1.03)	0.0019
Female	−0.39787	0.67 (0.58-0.78)	<0.0001
BMI category	−0.06847	0.93 (0.92-0.95)	<0.0001
NYHA functional class III or IV	0.20989	1.23 (1.07-1.43)	0.0051
CAD	−0.00532	1.00 (0.77-1.28)	0.97
COPD (moderate or severe)	0.45821	1.58 (1.31-1.91)	<0.0001
Hypertension	−0.13896	0.87 (0.74-1.02)	0.084
Insulin-dependent DM	0.22842	1.26 (0.94-1.68)	0.12
Carotid artery disease	−0.00587	0.99 (0.78-1.27)	0.96
Cerebrovascular disease	0.15691	1.17 (0.96-1.43)	0.13
Peripheral vessel disease	0.32908	1.39 (1.16-1.66)	0.0003
Malignancy	0.50462	1.66 (1.38-1.99)	<0.0001
Immunodeficiency	0.68142	1.98 (1.52-2.56)	<0.0001
Previous CABG	0.22975	1.26 (0.94-1.69)	0.13
Previous PCI	−0.10409	0.90 (0.70-1.17)	0.43
Pulmonary hypertension	0.04107	1.04 (0.88-1.23)	0.63
Porcelain aorta	0.3249	1.38 (1.14-1.69)	0.0013
Hemoglobin	−0.1201	0.89 (0.85-0.93)	<0.0001
Albumin	−0.46427	0.63 (0.56-0.71)	<0.0001
Creatinine	0.05308	1.06 (1.03-1.08)	0.0002
Aortic insufficiency grade III or IV at baseline	−0.18312	0.83 (0.66-1.06)	0.13
Mitral insufficiency grade III or IV at baseline	0.02473	1.03 (0.82-1.29)	0.83
Bicuspid aortic valve	−0.11631	0.89 (0.56-1.41)	0.62
LVEF per 1% increment	0.00217	1.00 (1.00-1.01)	0.44
Aortic valve mean gradient at baseline per 1 mm Hg increment	−0.01447	0.99 (0.98-0.99)	<0.0001
Aortic valve area at baseline per 1 cm^2^ increment	−0.15607	0.86 (0.65-1.12)	0.25
Nonelective procedure	0.57834	1.78 (1.27-2.50)	0.0008

Abbreviations as in Table 1.

### Validation of the model

**Figure 2:**
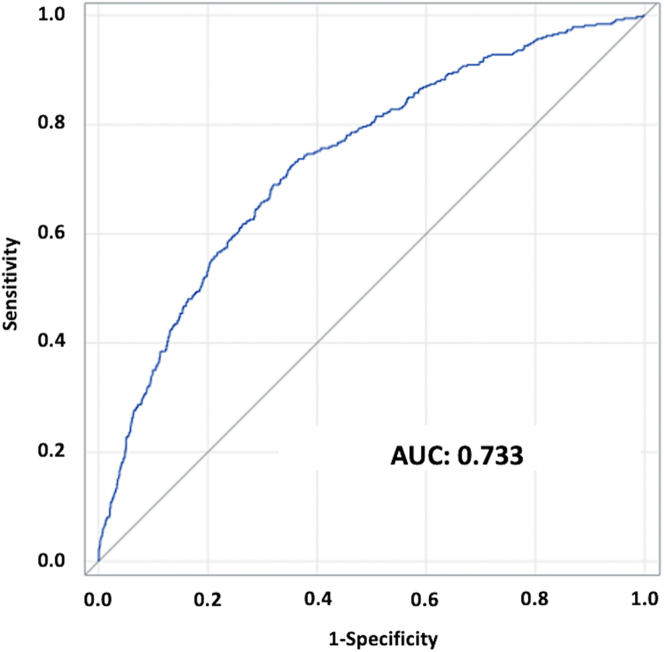
Time-Dependent Receiver-Operating Characteristic Curve A time-dependent receiver-operating characteristic curve at last event occurrence (day 364) estimated using the inverse probability of censoring weighting method showed good discrimination ability, with an area under the curve (AUC) of 0.733 (95% CI: 0.709-0.757).

**Figure 3:**
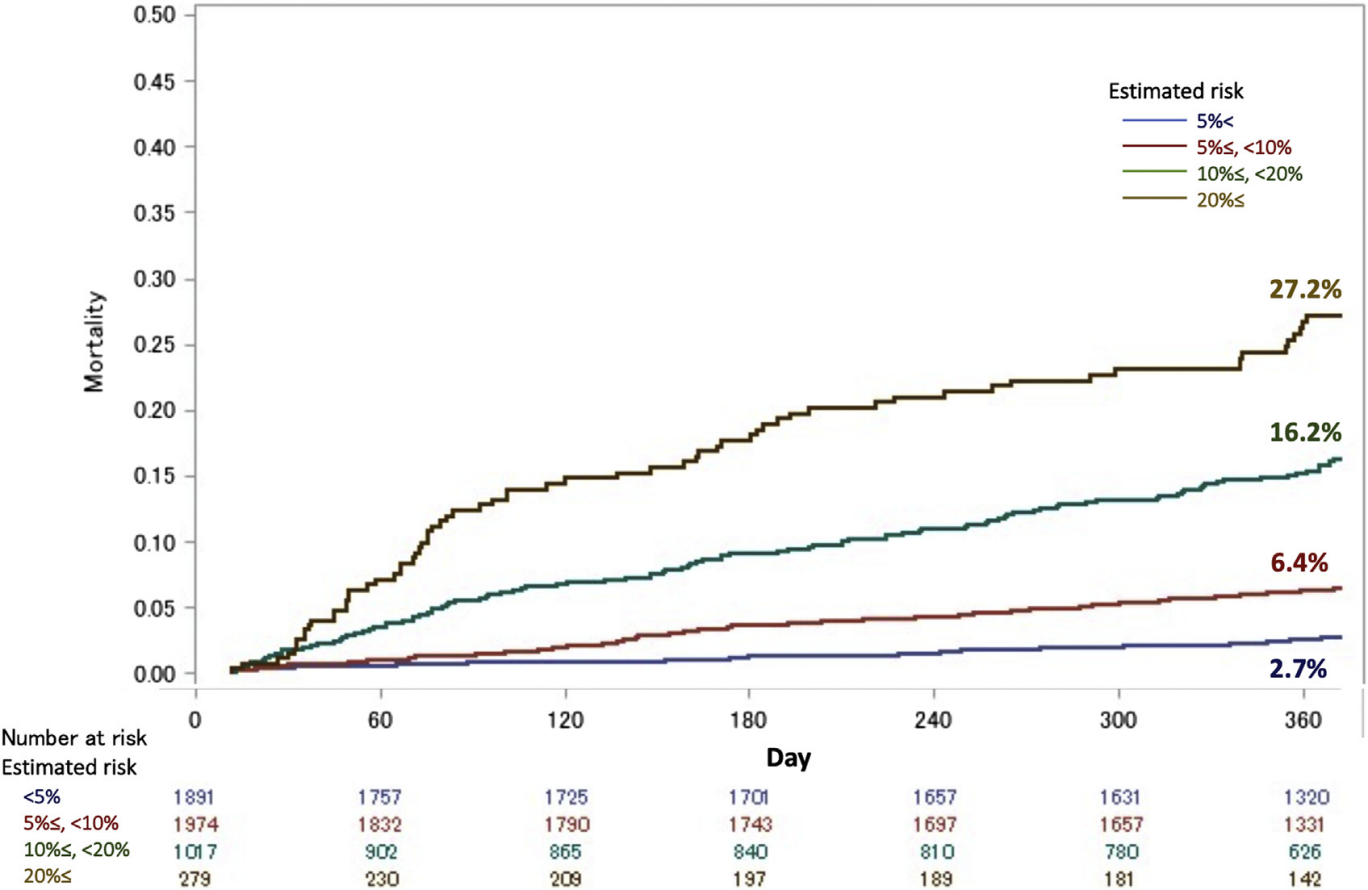
1-Year Mortality Among Patient Groups According to Estimated Mortality One-year survival rates correlated with estimated survival rates.

**Figure 4:**
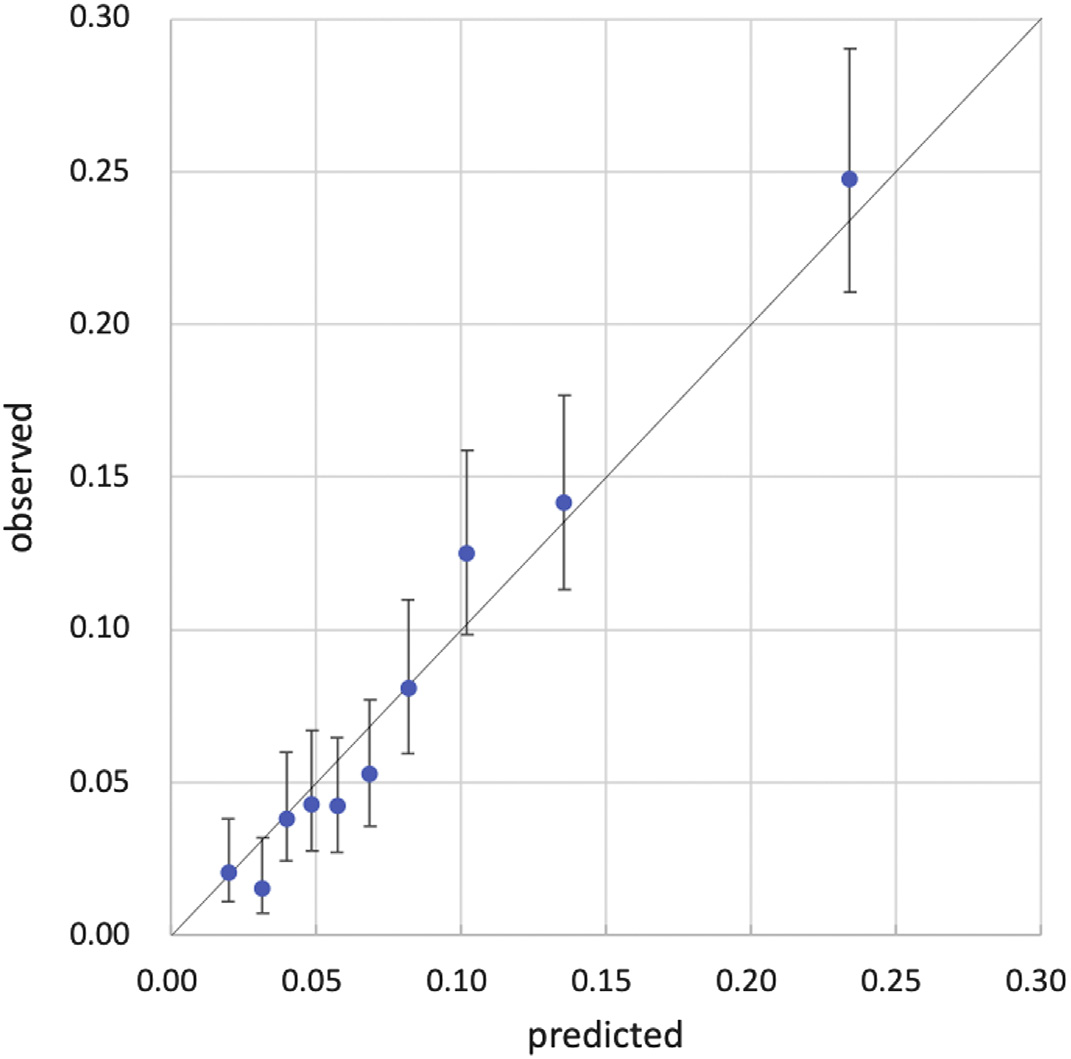
Calibration of the Abridged Model in the Validation Dataset When the validation dataset was divided into equally sized groups of 10 (on the basis of predicted mortality), the prediction model showed good calibration against observed mortality estimated using the Kaplan-Meier method.

Harrell’s C index in the validation model was 0.732 (95% CI: 0.712-0.753). The receiver-operating characteristic curve of this model is shown in Figure 2. The areas under the receiver-operating characteristic curves for the developed model, STS PROM score, and logistic EuroSCORE were 0.733 (95% CI: 0.709-0.757), 0.626 (95% CI: 0.597-0.655), and 0.599 (95% CI: 0.563-0.634), respectively (Supplemental Figure 1). Figure 3 shows the cumulative incidence function for 1-year mortality among 5 patient groups according to their predicted 1-year mortality probabilities in the validation cohort. Observed 1-year mortality rates were 2.7%, 6.4%, 16.2%, and 27.2% among patients with predicted mortality rates of <5%, 5% to <10%, 10% to <20%, and ≥20%, respectively. When dividing the patients in the validation cohort into 10 equally sized groups, the model showed good calibration between the predicted and observed mortality estimates from the Kaplan-Meier method (Figure 4), with a slight underestimation of risk in the lower risk groups.

### Additional analysis

The variable estimates from the multiple imputation analysis were quite similar to those from the primary analysis (Supplemental Table 2). The pooled C index from the 50 imputed validation cohorts was 0.730 (95% CI: 0.705-0.755).

## Discussion

We developed and validated the first predictive model (to the best of our knowledge) for assessing 1-year mortality risk following TAVR using national clinical data from the J-TVT registry. National clinical registries such as the TVT (Transcatheter Valve Therapy) Registry in the United States, FRANCE-2 (French Aortic National CoreValve and Edwards) registry, and UK TAVI (UK Transcatheter Aortic Valve Implantation) registry have become important resources to evaluate and improve clinical care and analyze the effectiveness of new treatment technologies. They provide information related to patients’ risk factors and outcomes, procedural and treatment trends, guideline adherence, and facility and provider characteristics. These registries also help researchers examine critical questions regarding treatment delivery and treatment outcomes.^7^^,^^18^, ^19^, ^20^, ^21^ Using these rich sources of real-world clinical information from the J-TVT registry, the statistical predictive model developed in this study demonstrated excellent calibration in the prediction of long-term mortality following TAVR. The C-index for STS PROM score was 0.644. The C-index at 1 year for the J-TVT registry model of 0.733 presented herein is better than the previously reported C index at 1 year for the STS PROM score of 0.56 and the EuroSCORE, also of 0.56, in a population from the PARTNER (Placement of Aortic Transcatheter Valve) trial following TAVR and its continued-access registry.^22^

The model developed in this study included parameters that were associated with poor prognosis after TAVR. Sex (male), procedural acuity, NYHA functional class III or IV, presence of chronic obstructive pulmonary disease, peripheral vessel disease, porcelain aorta, cancer, and high serum creatinine were previously reported to be predictors following TAVR.^7^^,^^12^^,^^13^^,^^23^^,^^24^ Furthermore, frailty is a well-known prognostic value after TAVR^12^^,^^20^^,^^25^, ^26^, ^27^ and is indispensable for developing risk models following TAVR. Frailty assessment includes various indexes.^28^ From the PARTNER trial, Green et al^26^ reported in the frail group increased mortality and a higher rate of poor outcomes 1 year after TAVR using 4 frailty markers (serum albumin, handgrip strength, gait speed, and Katz activities of daily living day). Kiani et al^20^ demonstrated that indexes of frailty including low albumin, anemia, and 5-m walk time, which were previously identified as markers of frailty available in the Society of Thoracic Surgeons/American College of Cardiology TVT Registry, are significantly associated with mortality at 30 days and 1 year. In this study, we included some of the identified markers (eg, serum albumin, hemoglobin). These variables may aid in the accurate assessment of comprehensive preoperative risk for frail patients.

Asian patients have smaller body habitus and aortic annular sizes and hence require smaller valves. These characteristics are usually associated with a higher risk for complications during the TAVR procedure.^29^ Our Japanese patients with AS in the J-TVT registry had smaller body habitus, with a mean BSA of 1.43 m^2^, compared with those in previously reported studies (BSA 1.7-1.8 m^2^, BMI 26.0 kg/m^2^).^20^^,^^29^ On the contrary, the present study showed a lower 30-day mortality rate (or in-hospital mortality) compared with registries from Western countries (5.3%-7.6%). Surprisingly, similar mortality rates have been achieved since 2013, when TAVR began in Japan.^14^ It is possible that such optimal outcomes resulted from a rigorous selection of qualified institutions and physicians, as well as manufacturers’ training. Moreover, TAVR was introduced in Japan when it was fully developed in Western countries. In support of this, at the onset of TAVR in Japan, health care providers routinely performed computed tomography–based (not echocardiography-based) screening, and this may have reduced the risk for complications that could have resulted from patients’ relatively smaller body sizes. However, Asian patients who undergo TAVR outside Western countries are scarcely included in the European registries or the TVT Registry and are underrepresented in clinical trials. Alkhouli et al^19^ reported lower 30-day and 1-year mortality in Asian, Native American, and Pacific Islander compared with White patients in the TVT Registry. However, they mentioned that these data should be interpreted with caution given the very small number of patients who underwent TAVR and the inclusion of multiple races in this group.

This study has several strengths. The uniqueness accorded by the J-TVT registry is the alignment of the data elements with the TVT Registry, conducted through the mutual activity related to Harmonization by Doing. Harmonization by Doing is a collaboration of industry, academia, and regulators between the United States and Japan that contributes to medical device development through the convergence of the evaluation and regulatory strategy. As the majority of TVT Registry and J-TVT data elements have been harmonized as a part of Harmonization by Doing, the data captured in these registries afford a rare opportunity to directly compare 2 international TAVR experiences. To the best of our knowledge, this model was developed from the largest number of Asian patients with AS and will be incorporated into the J-TVT registration system for clinical use. This should be applicable even to the occasional Asian patients encountered in Western countries.

### Study limitations

First, the present model enrolled patients undergoing TAVR who were mostly elderly, high-risk, or intermediate-risk patients treated between 2013 and 2018, which may not be representative of today’s populations and technologies, while remaining useful with respect to identifying patients in whom TAVR may be futile or even harmful. The use of new technologies and expansion of patient selection to a low-risk cohort may reveal better long-term outcomes. Therefore, our model should be prospectively evaluated for its applicability to today’s populations.

Second, the generalizability of our model to populations other than those undergoing TAVR in Japan is unknown. As the development and validation of the model were performed in randomly selected subsamples of the same patient population, external validation is needed for the assessment of its applicability to other patient populations.

Third, in terms of morbidity after TAVR, as J-TVT was originally designed to capture early or perioperative severe and nonsevere adverse events directly related to the TAVR procedure rather than those in the remote phase as in the present study, adjudication of adverse outcomes after index hospitalization in the remote phase remains a challenge.

Finally, given the characteristics of the elderly patients who are generally considered for TAVR, understanding the health status outcomes of these procedures is essential. The TVT Registry has been working with the Centers for Medicare and Medicaid Services to evaluate postoperative quality of life among patients using the Kansas City Cardiomyopathy Questionnaire overall summary score. This is expected to contribute to improve patient selection for future TAVR.^30^ Although the current J-TVT registry does not include an item to assess postoperative quality of life, more granular categorical analyses of patients’ overall condition provide further perspectives on the effect of these interventions over time. Providing clinically meaningful outcomes in standardized definitions remains a main goal of our consortium.

## Conclusions

Using 12,316 patients for the derivation cohort and 5,339 patients for the validation cohort from the J-TVT registry from 2013 to 2018, we have developed and validated predictive models for assessing 1-year mortality risk following TAVR. This is the first predictive model for 1-year mortality following TAVR derived from a national clinical database, and it demonstrates good calibration. The model should aid physicians in identifying patients in whom TAVR may be futile or even harmful (Central Illustration).

Perspectives **COMPETENCY IN MEDICAL KNOWLEDGE:** A well-accepted statistical 1-year risk model specifically designed for the population undergoing TAVR is a foundational element for several reasons. First, any intervention to correct an AS lesion (TAVR and surgical aortic valve replacement) is recommended for patients who would benefit from it in the long term and are expected to survive beyond 12 months, from the perspective of therapeutic effect and/or cost-effectiveness. Second, further knowledge of late outcomes can assist physicians and patients in making informed decisions.

**TRANSLATIONAL OUTLOOK:** As the majority of TVT Registry and J-TVT data elements have been harmonized, the data captured in these registries afford a rare opportunity to directly compare 2 international TAVR experiences. Therefore, we expect future studies to apply this model to the Asian cohort in the TVT Registry to identify the utility of this model in the United States.

**Central Illustration:**
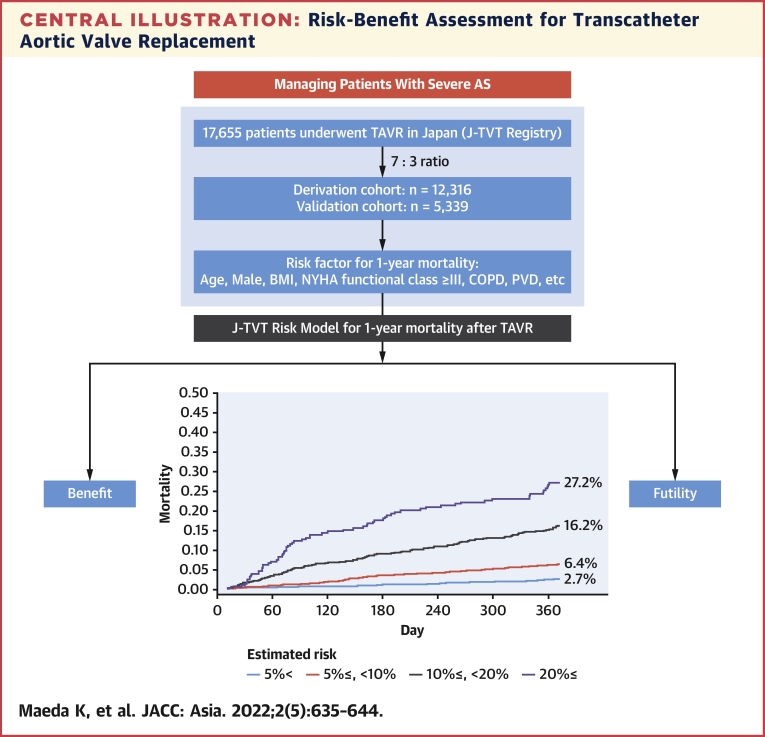
Risk-Benefit Assessment for Transcatheter Aortic Valve Replacement The J-TVT (Japan Transcatheter Valve Therapies) risk model is useful in identifying patients who may or may not benefit from transcatheter aortic valve replacement (TAVR) because it predicts 1-year outcomes on the basis of preoperative factors. AS = aortic valve stenosis; BMI = body mass index; COPD = chronic obstructive pulmonary disease; NYHA = New York Heart Association; PVD = peripheral vascular disease.

## Funding Support and Author Disclosures

The authors have reported that they have no relationships relevant to the contents of this paper to disclose.
