# Supplementary material for: A Risk Model for 1-Year Mortality After Transcatheter Aortic Valve Replacement From the J-TVT Registry
Source: JACC Asia. 2022 Oct 4;2(5):635–44. doi: 10.1016/j.jacasi.2022.06.002 (PMC9743452; doi:10.1016/j.jacasi.2022.06.002)

Supplemental Table 1. Additional characteristics of the patients (the mean STS score, timing of cohort entry, and 2^nd^ and 3^rd^ generation device use) in the derivation and validation cohorts.

|  |  | derivation | validation |
| --- | --- | --- | --- |
| N |  | 12316 | 5339 |
| STS score, % (mean ± SD) | | 7.3 ± 4.9 | 7.4 ± 4.7 |
| cohort entry, no. of pts (%) | |  |  |
|  | 2013 to 2014 | 698 (5.7) | 341 (6.4) |
|  | 2015 to 2016 | 3306 (26.8) | 1444 (27.0) |
|  | 2017 to 2018 | 8312 (67.5) | 3554 (66.6) |
| Device, no. of pts (%) | |  |  |
| 2nd generation  (SAPIEN XT/CoreValve) | | 3548 (28.8) | 1592 (29.8) |
| 3rd generation  (SAPIEN3/Evolut R/Evolut PRO) | | 8744 (71.0) | 3733 (69.9) |

Supplemental Table 2. Estimates from the multivariable Cox proportional hazards regression analysis using 50 multiple imputed datasets for 1-year mortality after transcatheter aortic valve replacement

| Variable | Coefficient | HR (95% confidence interval) | p-value |
| --- | --- | --- | --- |
| Age | 0.021789 | 1.02 (1.01-1.04) | 0.0011 |
| Gender (Female) | -0.38728 | 0.68 (0.59-0.78) | <.0001 |
| BMI category | -0.06864 | 0.93 (0.92-0.95) | <.0001 |
| NYHA III or IV | 0.232567 | 1.26 (1.09-1.46) | 0.0016 |
| CAD | -0.07112 | 0.93 (0.72-1.20) | 0.58 |
| COPD (moderate or severe) | 0.446314 | 1.56 (1.29-1.89) | <.0001 |
| Hypertension | -0.14246 | 0.87 (0.74-1.01) | 0.073 |
| Insulin-dependent DM | 0.218472 | 1.24 (0.93-1.66) | 0.14 |
| Carotid artery disease | 0.001223 | 1.00 (0.78-1.28) | 0.99 |
| Cerebrovascular disease | 0.145729 | 1.16 (0.94-1.42) | 0.16 |
| Peripheral vessel disease | 0.352677 | 1.42 (1.19-1.70) | <.0001 |
| Malignancy | 0.516824 | 1.68 (1.39-2.02) | <.0001 |
| Immunodeficiency | 0.677449 | 1.97 (1.52-2.55) | <.0001 |
| Previous CABG | 0.277843 | 1.32 (0.99-1.77) | 0.062 |
| Previous PCI | -0.05414 | 0.95 (0.73-1.22) | 0.68 |
| Pulmonary hypertension | 0.037813 | 1.04 (0.88-1.23) | 0.66 |
| Porcelain aorta | 0.31848 | 1.38 (1.13-1.67) | 0.0014 |
| Hemoglobin | -0.13208 | 0.88 (0.84-0.91) | <.0001 |
| Albumin | -0.38269 | 0.68 (0.60-0.77) | <.0001 |
| Creatinine | 0.053141 | 1.05 (1.03-1.08) | 0.0002 |
| Aortic insufficiency III or IV at baseline | -0.17779 | 0.84 (0.66-1.06) | 0.14 |
| Mitral insufficiency III or IV at baseline | 0.034623 | 1.04 (0.83-1.30) | 0.76 |
| Bicuspid aortic valve | -0.11321 | 0.89 (0.56-1.42) | 0.63 |
| LVEF per 1% increment | 0.001331 | 1.00 (1.00-1.01) | 0.63 |
| Aortic valve mean gradient at baseline per 1 mmHg increment | -0.01425 | 0.99 (0.98-0.99) | <.0001 |
| Aortic valve area at baseline per 1 cm^2^ increment | -0.16605 | 0.85 (0.65-1.11) | 0.23 |
| Non-elective procedure | 0.65601 | 1.93 (1.40-2.66) | <.0001 |

BMI, body mass index; NYHA, New York Heart Association; CAD, coronary artery disease; COPD, chronic obstructive pulmonary disease; DM, diabetes mellitus; CABG, coronary artery bypass grafting; PCI, percutaneous coronary intervention; LVEF, left ventricular ejection fraction.

Supplemental Figure 1. The ROC curves for STS score and logistic euroSCORE among the patients in the validation cohort.


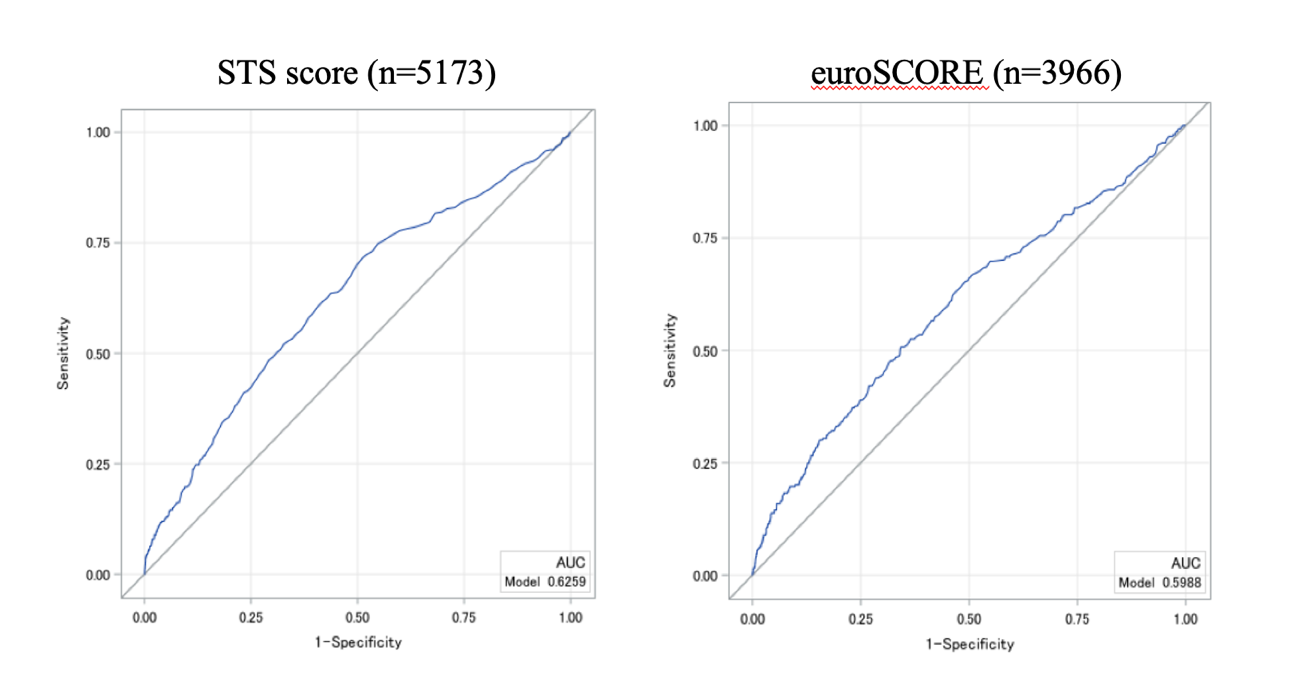

Supplement: Supplemental Tables 1 and 2 and Supplemental Figure 1 [file mmc1.docx]
